# Dosimetric comparison of the shoulder region between paired photon and proton plans in breast cancer patients

**DOI:** 10.1016/j.tipsro.2026.100393

**Published:** 2026-03-22

**Authors:** Yuqin Liang, Karolien Verhoeven, Gloria Vilches-Freixas, Sophie C.J. Bosma, Richard Canters, Femke E. Froklage, Ruud Houben, John H. Maduro, Jelle Overbosch, Joan J. Penninkhof, Margriet G.A. Sattler, Janine M. Simons, Nanna M. Sijtsema, Rozemarijn Vliegenthart, Anne P.G. Crijns, Sofia Rivera, Liesbeth J. Boersma

**Affiliations:** a Department of Radiation Oncology (Maastro), GROW-Research Institute for Oncology and Reproduction, Maastricht University Medical Centre+, Maastricht, the Netherlands; b Department of Radiation Oncology, Leiden University Medical Center, Leiden, the Netherlands; c Department of Radiotherapy, Erasmus MC Cancer Institute, University Medical Center Rotterdam, Rotterdam, the Netherlands; d Department of Radiation Oncology, University Medical Center Groningen, University of Groningen, Groningen, the Netherlands; e Department of Radiology, University Medical Center Groningen, University of Groningen, Groningen, the Netherlands; f Holland PTC, Holland PTC, Delft, the Netherlands; g Radiation Oncology Department, Gustave Roussy, Villejuif, France

## Abstract

**Background:**

Adjuvant radiotherapy lowers regional recurrence in breast cancer but may cause arm symptoms. We compared photon (XRT) and proton therapy (PT) doses to eight shoulder structures, including seven muscles and the axillary–lateral thoracic junction (ALTJ).

**Methods:**

We consecutively included all patients treated with PT in our centre for whom paired XRT–PT plans (2019–2024) were available. Eight shoulder structures were autodelineated with manual correction of the ALTJ region. For each parameter, medians (Q1–Q3) were calculated, and paired differences were defined as Δᵢ = XRTᵢ − PTᵢ and summarized by the median of Δᵢ for subject i; Statistical inference used the Wilcoxon signed-rank test with Hodges–Lehmann estimates (95% confidence intervals). Multiple comparisons were adjusted using the Benjamini–Hochberg procedure. Exploratory subgroup analyses were stratified by Regional nodal irradiation (RNI) levels. Analyses were performed in R (version 4.5.2).

**Results:**

In 128 paired plans, XRT delivered significantly higher doses than PT to all shoulder muscles. For the five key muscles, XRT delivered 10.0–21.1 Gy higher D_mean_ and 29.6–64.6% higher V_15Gy_, indicating a high advantage for PT. A significant moderate advantage was observed for the latissimus dorsi, with minimal yet significant for the trapezius and ALTJ. Subgroup analyses further showed that XRT delivered significantly higher doses than PT to the five key muscles in patients receiving RNI at levels III–IV or I–IV.

**Conclusion:**

PT offers substantial dosimetric advantages for five shoulder muscles compared with XRT. The clinical relevance of these dosimetric differences requires further study.

## Highlights

• First proton–photon dosimetric comparison of the ALTJ region in breast cancer.
• Proton therapy delivered lower doses to five major shoulder muscles than photon did.
• Proton therapy reduced ALTJ dose when levels I–II axillary radiotherapy were omitted.

## Introduction

Breast cancer (BC) treatment typically includes surgery, systemic therapy, and radiotherapy, depending on tumor biology and stage. Radiotherapy improves locoregional control and overall survival [1], [2], especially in patients with nodal involvement. However, adjuvant radiotherapy can lead to arm and shoulder symptoms (AS) in 6–30% of patients [3], [4], [5], including pain, lymphedema, and reduced shoulder function, significantly impacting health-related quality of life [6].

Common risk factors for lymphedema after BC treatment include axillary lymph node dissection (ALND), a higher number of lymph nodes removed, and elevated body mass index (BMI) [7], [8], [9], [10], [11]. Adjuvant radiotherapy is also an independent risk factor for both lymphedema [11] and overall AS [12]. Building on this, recent studies have begun examining which anatomical regions receive higher radiotherapy doses and how this contributes to AS risk [13], [14], [15], [16], [17], [18].

The axillary-lateral thoracic vessel juncture (ALTJ) region has been identified as a structure associated with increased risk of arm lymphedema [13]. D_min_ ≥ 36.8 Gy to the ALTJ, a higher number of lymph nodes removed, and higher BMI significantly increased the risk of lymphoedema, with hazard ratios (HRs) of 7.69 (D_min_ ≥ 36.8 Gy versus < 36.8 Gy), 1.08 per node, and 1.06 per unit, respectively. Over the past five years, several studies have explored the link between radiation dose to the ALTJ—specifically D_max_, D_mean_ and V_35Gy_ [%] and lymphedema development. While most suggest a potential association [13], [16], [17], [18], one study found no significant correlation [14], highlighting ongoing inconsistency in the evidence.

Other studies have analysed shoulder structures by assessing radiation dose to the global shoulder region [15] and individual shoulder muscles [19], [20], [21]. Johansen *et al.* analysed the relationship between dose to global shoulder structures and clinical AS occurrence [15]. The Shoulder volume was defined from the humeral head to the acromion and coracoid process, with a 0.5 cm margin to include nearby soft tissue and the acromioclavicular joint. Increased V_15Gy_ in this volume was correlated to higher risk of lymphedema and reduced shoulder abduction [15]. Subsequently, Lipps *et al.* shifted focus from the global shoulder volume to individual muscles. Their dosimetric analysis showed that the mean dose (D_mean_) the pectoralis major and minor, latissimus dorsi, and teres major varied across different radiation field arrangements [19].

Since proton therapy (PT) is known to deliver lower doses to organs at risk (OAR), it could be expected that PT would also result in lower doses, and consequently a lower risk of AS, compared with photon therapy (XRT) [22]. However, there are very limited data on dosimetric differences in the shoulder region between XRT and PT plans [20], [21]. Prior small-cohort studies (n = 5 and n = 30) reported substantial dose reductions with PT, with V_30Gy_ decreased by 25–82% in six posterior shoulder muscles [21] and demonstrated the largest differences for low- and intermediate-dose metrics (V_15Gy_ and D_50cc_) in the trapezius, latissimus dorsi, and glenohumeral joint [20].

The aim of this study was to compare dose differences across eight shoulder structures between XRT and PT in a larger real-world clinical cohort, including seven muscles and the previously unstudied ALTJ region.

## Methods

### Study design

This retrospective paired analysis was approved by the Institutional Review Board (IRB) of the study centre (Affiliation 1). A waiver of informed consent was granted under the Dutch Medical Treatment Contracts Act (WGBO), which allows data use for public-interest research if patients have not objected.

### Patients selection

In this study, BC patients were selected for PT to reduce the risk of acute coronary events and secondary lung or breast cancers, following national indication protocols [23], [24]. We included consecutive patients treated with PT at a single centre (2019–2024) with both plans available. Eligible patients received irradiation to the breast, chest wall, and/or regional lymph nodes, with or without a boost.

### Radiotherapy details

#### Simulation

The patient was positioned supine with arms raised for both scans. For right-sided BC, free breathing (FB) was standard for both PT and XRT, typically using the same non-contrast CT scan. For left-sided cases, FB was used for PT and voluntary moderate deep inspiration breath hold (vmDIBH) for XRT, with consecutive CT scans under the same setup.

#### Targets volumes & dose

Target volumes were delineated per ESTRO guidelines [25]. The elective clinical target volume (CTV_elective_) included the whole breast or chest wall, with or without regional lymph nodes—axillary (levels I–IV), periclavicular (III–IV), and optionally internal mammary nodes (IMN). The boost clinical target volume (CTV_boost_) targeted the tumor bed or a specific lymph node based on clinical indication [26]. In XRT, planning target volumes (PTVs) were generated by adding a uniform 5 mm margin to clinical target volumes (CTVs), cropped 5 mm inside the skin for dose evaluation. Both XRT and PT used the same prescription: 2.67 Gy per fraction. PT doses were taken from the nominal plan and reported as Gy (RBE), assuming RBE = 1.1 for comparison with XRT.

#### Technique & fields

Treatment plans for XRT and PT were created using our standard clinically applied techniques: XRT used a hybrid technique combining volumetric-modulated arc therapy and three-dimensional conformal radiotherapy (Hybrid VMAT-3DCRT) approach to cover the PTVs, delivering approximately 80% of the dose with tangents and the rest via a volumetric arc to the breast and regional lymph nodes [26], [27]. PT plans were robustly optimized for CTV_elective_ and CTV_boost_, with ± 5 mm setup and ± 3% range uncertainty [28], typically using 2–5 fields, including at least one en face field perpendicular to the chest wall.

#### Plan evaluation

In routine practice, dose coverage criteria differed slightly between XRT and PT. For XRT, at least 98% of the PTV had to receive ≥ 95% of the prescribed dose (V_95%_ ≥ 98%), with a mean PTV dose (D_mean_) between 99% and 101% [29]. For PT, target coverage had to meet V_93%_ of the CTV ≥ 98% in the voxel-wise minimum dose plan, which correlates with the PTV constraint used in XRT [30]. OAR constraints for XRT and PT were similar [29], [31].

### Automatic segmentation of shoulder structures

An atlas-based segmentation approach was used to facilitate the delineation of shoulder muscles and improve workflow efficiency. All automatically generated contours were subsequently reviewed and adjusted by an experienced radiation oncologist to ensure anatomical accuracy and consistency across patients. Examples of shoulder region delineation are shown in Supplementary Figs. S1–S2.

### Recorded dosimetric data

For the shoulder muscles, we calculated V_15Gy_ [%] [15] and D_mean_ [Gy] [19]. For the ALTJ region, we assessed D_mean_ [Gy] [16], D_max_ [Gy] [18], D_min_ [Gy] [13] and V_35Gy_ [%] [17]. Vx [%] refers to the percentage of a volume receiving at least x Gy of radiation dose. For target coverage (CTV_elective_ and CTV_boost_), we evaluated D_2%_ [Gy] (dose to the hottest 2% of the volume; maximum-dose proxy), D_98%_ [Gy] (dose to 98% of the volume; minimum-dose proxy), and D_mean_ [Gy] (mean dose), all in Gy. For each parameter, Δ_i_ denotes the paired difference for subject *i*, defined as XRT_i_ − PT_i_; positive values indicate a dosimetric advantage for PT. Values are reported as the median of Δ_i_. The degree of advantage was classified into four categories: high, moderate, low, or no advantage (see Table 1).

**Table 1:** median-based criteria for dosimetric differences in shoulder muscles and the ALTJ region

Category	Criteria for shoulder muscles*	Criteria for ALTJ†	p (BH)‡
High advantage	median(Δ_i_) ≥ 10 Gy for D_mean_**and** ≥ 10% for V_15Gy_	median(Δ_i_) ≥ 10 Gy for D_min_, D_mean_, or D_max_, **or** ≥ 10% for V_35Gy_	< 0.05
Moderate advantage	median(Δ_i_) ≥ 5 to < 10 Gy for D_mean_**and** ≥ 5 to < 10% for V_15Gy_	median(Δ_i_) ≥ 5 to < 10 Gy for D_min_, D_mean_, or D_max_, **or** ≥ 5 to < 10% for V_35Gy_	< 0.05
Low advantage	median(Δ_i_) > 0 to < 5 Gy for D_mean_**and** > 0 to < 5% for V_15Gy_	median(Δ_i_) > 0 to < 5 Gy for D_min_, D_mean_, or D_max_, **or** > 0 to < 5% for V_35Gy_	< 0.05
No advantage	Does not meet above thresholds	Does not meet above thresholds	≥ 0.05

Abbreviations: **ALTJ**, axillary–lateral thoracic vessel juncture; **p (BH),** Benjamini–Hochberg adjusted p value; **median (Δ**_i_**)**, median of within-patient paired differences for each parameter, where Δ_i_ = XRT_i_ − PT_i_ for subject i; **D_mean_ [Gy]**, mean dose; **D_min_ [Gy]**, minimum dose; **D_max_ [Gy]**, maximum dose; **V_15Gy_ [%]**, percentage of the structure volume receiving ≥15 Gy; **V_35Gy_ [%]**, percentage of the structure volume receiving ≥ 35 Gy.

* For the shoulder muscles, both D_mean_ [Gy] and V_15Gy_ [%] were required to meet the thresholds, as they represent the low- to intermediate-dose range. If absolute and relative differences fell into different categories, the lower category was assigned.

† For the ALTJ region, thresholds across different dose levels (low, intermediate, and high) were applied independently; fulfilling either the absolute or the relative criterion was sufficient.

‡ Based on p (BH), p < 0.05 was considered statistically significant.

### Statistical analysis

For each parameter, medians and interquartile ranges (Q1–Q3) for XRT and PT were calculated using complete pairs. Paired differences were defined as Δ_i_ = XRT_i_ − PT_i_ and descriptively summarized by the median of Δ_i_ to reflect the typical patient-level difference. Statistical inference was performed using the Wilcoxon signed-rank test, and the corresponding p values are reported as p (paired). Effect sizes were quantified using Hodges–Lehmann (HL) estimates with 95% confidence intervals (CIs), capturing the overall directional shift between XRT and PT. The primary analysis included the overall cohort, while subgroup analyses by target volume were considered exploratory. To account for multiple comparisons, p (paired) values were adjusted using the Benjamini–Hochberg procedure to control the false discovery rate at 5%, and adjusted p values are reported as p (BH). Statistical significance was defined as p (BH) < 0.05. Parameters with fewer than five paired observations or with zero variance were excluded. All analyses were performed in R (version 4.5.2).

## Results

### Patient Inclusion

**Fig. 1:**
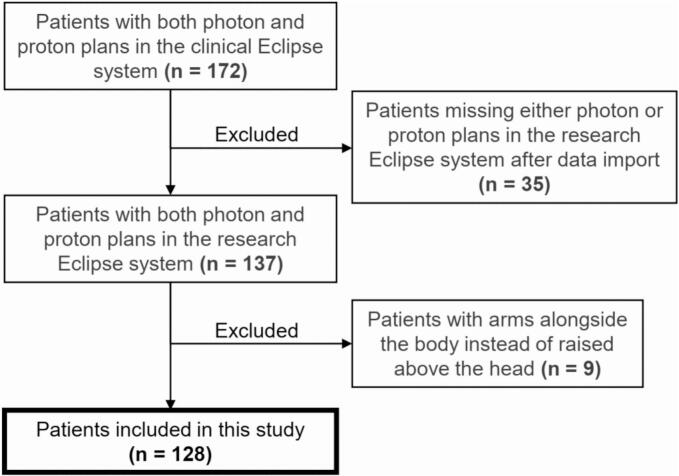
Flowchart of patient inclusion.

Initially, 172 patients treated in PT with both XRT and PT plans in Eclipse were identified. After excluding 35 due to failed plan import and 9 due to arms-down positioning, 128 patients remained for analysis (127 unilateral, 1 bilateral BC; see Fig. 1).

### Radiotherapy Characteristics

Of the 128 patients, 65 (50.8%) received regional nodal irradiation (RNI) to levels I–IV, 31 (24.2%) to levels III–IV only, 8 (6.3%) to levels I–II only, and 24 (18.8%) had no levels III–IV. IMN irradiation was delivered in 84 patients (65.6%). All patients received at least 40.05 Gy in 15 fractions. Among 55 patients who received a boost, 44 were treated with 53.4 Gy in 20 fractions, and 11 received a dose-escalated boost (58.74 Gy in 22 fractions, n = 3; 61.41 Gy in 23 fractions, n = 8). Boost target details are shown in Supplementary Table A.

### Target volume coverage

Both XRT and PT plans met the pre-specified coverage criteria for CTV_elective_, CTV_boost_ and all RNI subsites, with no clinically meaningful differences (Supplementary Table B). Volumetric analysis showed significant but clinically irrelevant differences between XRT and PT in the full cohort (n = 128) and boost subset (n = 55). XRT using breath-hold CT produced slightly larger volumes than PT using free-breathing CT, with a significant difference for the breast (median(Δ_i_) = 19.0 cc; p = 0.002) but not for the chest wall (median(Δ_i_) = 17.1 cc; p = 0.065).

### Overall dosimetric analysis

**Fig. 2:**
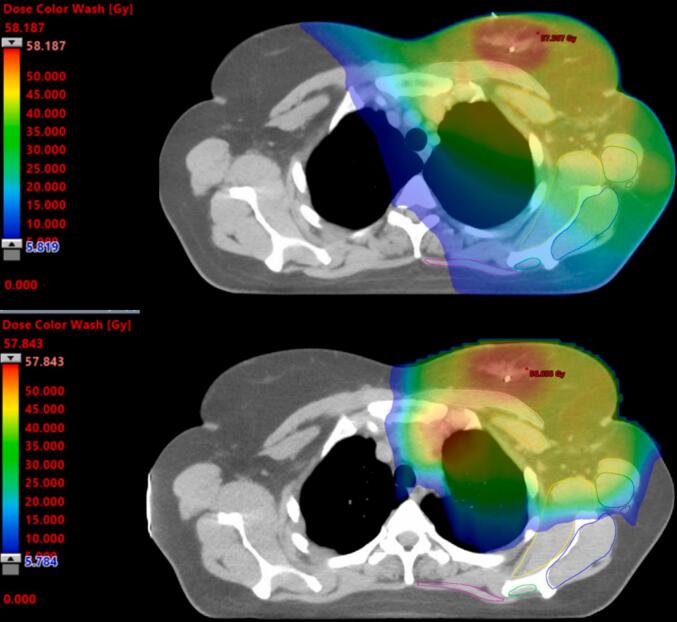
Example of axial dose distribution for photon therapy (XRT, top) and proton therapy (PT, bottom).

An example of axial dose distribution comparing XRT (top) and PT (bottom) is shown in Fig. 2, illustrating reduced dose to the shoulder muscles with PT. Below, we described which structures had a high, moderate or low advantage of PT compared to XRT, respectively.

#### High advantage

Across the cohort (Table 2), XRT delivered substantially higher doses than PT to the five shoulder muscles (teres major, teres minor, supraspinatus, infraspinatus, and subscapularis). Descriptively, the median paired differences [median(Δ_i_)] indicated that XRT was associated with 10.0–21.1 Gy higher D_mean_ and 29.6–64.6% higher V_15Gy_ compared with PT, meeting the criteria for a high dosimetric advantage for PT. Paired comparisons based on HL estimates demonstrated a consistent overall shift between XRT and PT that remained statistically significant after adjustment for multiple testing (p(BH) < 0.001).

**Table 2:** Dosimetric comparison of individual shoulder regions between photon (XRT) and proton (PT) plans (n = 128).

**Dosimetric parameters**	**XRT median (IQR)**	**PT median (IQR)**	**median (Δ**_i_**)**	**HL shift (95% CI)**	**p (paired)**	**p (BH)**
**Teres major D_mean_ [Gy]**	23.5 (18.9–26.9)	10.2 (2.4–14.9)	12.4	12.64 (11.52 to 13.76)	<0.001	<0.001
**Teres major V_15Gy_ [%]**	68.7 (59.5–74.8)	21.4 (0–43.8)	36.9	41.09 (37.05 to 45.35)	<0.001	<0.001
**Teres minor D_mean_ [Gy]**	22.6 (13.5–31.3)	1 (0.3–1.9)	21.1	20.20 (18.17 to 22.33)	<0.001	<0.001
**Teres minor V_15Gy_ [%]**	64.9 (35.2–95)	0 (0–0)	64.6	66.46 (59.48 to 72.79)	<0.001	<0.001
**Supraspinatus D_mean_ [Gy]**	16.6 (9.9–19.2)	0 (0–0.2)	16.4	15.07 (12.32 to 16.53)	<0.001	<0.001
**Supraspinatus V_15Gy_ [%]**	61.6 (2.8–75.5)	0 (0–0)	60.9	66.07 (61.99 to 69.53)	<0.001	<0.001
**Infraspinatus D_mean_ [Gy]**	13.2 (8.6–16.4)	0.2 (0.1–0.6)	12.6	12.31 (11.18 to 13.47)	<0.001	<0.001
**Infraspinatus V_15Gy_ [%]**	39.5 (22.4–52.3)	0 (0–0)	39.5	41.12 (37.34 to 45.00)	<0.001	<0.001
**Subscapularis D_mean_ [Gy]**	19.9 (15.1–23.5)	8.8 (3.4–12.2)	10.0	10.11 (9.05 to 11.26)	<0.001	<0.001
**Subscapularis V_15Gy_ [%]**	57.8 (42.1–69.1)	23.8 (6.5–33.8)	29.6	31.57 (27.73 to 35.60)	<0.001	<0.001
**Latissimus dorsi D_mean_ [Gy]**	13 (10.3–16)	7.5 (4.7–10.3)	5.0	5.20 (4.33 to 6.08)	<0.001	<0.001
**Latissimus dorsi V_15Gy_ [%]**	32.8 (25.9–41.8)	0 (0–0)	30.5	31.29 (28.54 to 33.96)	<0.001	<0.001
**Trapezius D_mean_ [Gy]**	5.7 (3.3–7.3)	0 (0–0.2)	5.5	5.20 (4.51 to 5.77)	<0.001	<0.001
**Trapezius V_15Gy_ [%]**	5 (0–15.4)	0 (0–0)	4.9	11.20 (9.43 to 13.19)	<0.001	<0.001
**ALTJ D_max_ [Gy]**	42 (41.1–45.4)	42.4 (41.8–45.8)	-0.2	-0.12 (-0.41 to 0.23)	0.485	0.485
**ALTJ D_mean_ [Gy]**	39.5 (32.2–40.4)	39.2 (27.5–41)	0.1	0.95 (0.31 to 2.05)	0.002	0.003
**ALTJ D_min_ [Gy]**	24.4 (13–36.2)	22.9 (4.5–32.1)	3.1	4.51 (2.80 to 5.92)	<0.001	<0.001
**ALTJ V_35Gy_ [%]**	95.5 (36–100)	88.1 (29.4–99.8)	0.0	2.32 (0.49 to 5.25)	0.008	0.008

Abbreviations: **ALTJ**, axillary–lateral thoracic vessel juncture; **XRT**, photon therapy; **PT**, proton therapy; **D_mean_ [Gy]**, mean dose; **D_min_ [Gy]**, minimum dose; **D_max_ [Gy]**, maximum dose; **V_15Gy_ [%]**, percentage of the structure volume receiving ≥15 Gy; **V_35Gy_ [%]**, percentage of the structure volume receiving ≥35 Gy; **XRT median [IQR],** median and interquartile range (Q1–Q3) for photon therapy; **PT median [IQR],** median and interquartile range (Q1–Q3) for proton therapy; HL shift (95% CI), Hodges–Lehmann estimate of the paired difference (XRT − PT) with 95% confidence interval; **median (Δ**_i_**)**, median of within-patient paired differences for each parameter, where Δ_i_ = XRT_i_ − PT_i_ for subject i; **p (paired),** Wilcoxon signed-rank test; **p (BH),** Benjamini–Hochberg adjusted p value. Definitions of dosimetric advantage for proton therapy compared with photon therapy are described in Table 1 and the corresponding footnote.

#### Moderate advantage

For the latissimus dorsi (Table 2), XRT delivered higher doses than PT. Descriptively, the median paired differences [median(Δ_i_)] indicated that XRT was associated with 5.0 Gy higher D_mean_ and 30.5% higher V_15Gy_ compared with PT, meeting the criteria for a moderate dosimetric advantage for PT. Paired comparisons based on HL estimates demonstrated a statistically significant overall shift between XRT and PT after adjustment for multiple testing (p(BH) < 0.001).

#### Low advantage

For the trapezius (Table 2), XRT delivered higher doses than PT. The median paired differences [median(Δ_i_)] showed a 5.5 Gy higher D_mean_ and a 4.9% higher V_15Gy_ with XRT, corresponding to a low dosimetric advantage for PT. Paired comparisons based on HL estimates confirmed a statistically significant overall shift after multiple-testing adjustment (p(BH) < 0.001).

For the ALTJ region (Table 2), XRT also delivered higher doses than PT.

Descriptively, only D_min_ showed a median paired difference [median(Δ_i_)] of 3.1 Gy higher with XRT, corresponding to a low dosimetric advantage for PT. In contrast, median paired differences for D_mean_, V_35Gy_, and D_max_ were negligible (median(Δ_i_) = 0.1, 0, and -0.2, respectively). Paired comparisons based on HL estimates demonstrated statistically significant overall differences for D_min_, D_mean_, and V_35Gy_ after adjustment for multiple testing (p(BH) < 0.001, 0.003, and 0.008, respectively), whereas the difference for D_max_ was not statistically significant (p(BH) = 0.485).

### Subgroup analysis

Across RNI subgroups, XRT delivered higher doses than PT, with the magnitude of the difference increasing with the extent of the RNI field. In patients treated to RNI levels III–IV or I–IV, XRT was associated with 10.5–23.8 Gy higher D_mean_ and 30.3–73.6% higher V_15Gy_, corresponding to a high dosimetric advantage for PT.

In patients treated to levels I–II only, a high advantage for PT persisted for the teres major and teres minor, with XRT delivering 12.2–19.0 Gy higher D_mean_ and 28.8–71.0% higher V_15Gy_. Among patients without nodal irradiation to levels I–IV, significantly higher doses with XRT were observed only for the teres major (13.9 Gy higher D_mean_ and 38.1% higher V_15Gy_), also indicating a high advantage for PT.

For the ALTJ, PT demonstrated a moderate advantage, with 8.0 Gy lower D_mean_ in patients without RNI and 9.4 Gy lower D_min_ in those treated to levels III–IV. Detailed subgroup results for all structures including multiple comparison are provided in Supplementary Table C.

## Discussion

This study shows that PT significantly reduces radiation dose to the shoulder region compared to XRT. Dose reductions were observed across all seven evaluated muscles, with the greatest benefit in the five muscles closest to the CTV. PT provided limited dosimetric advantage for the ALTJ region in the overall cohort analysis. These findings suggest that PT may be associated with improved preservation of upper limb mobility and a reduced risk of AS [15].

Our study builds on previous work while introducing several methodological differences. Compared with Burlile *et al.* [20], we analysed a cohort that included 104 patients who received RNI to axillary levels I–II and/or III–IV and 24 patients who received no irradiation to levels I–IV. All patients received a uniform fractionation regimen of 2.67 Gy and were treated in an arms-up position. We also used atlas-based segmentation, which has the potential to reduce contouring time in large patient cohorts and improve workflow efficiency. XRT were generated with a Hybrid VMAT-3DCRT technique to optimize cardiac and pulmonary sparing while maintaining robust CTV coverage [26], [27]. Additional differences are noted when comparing with Prescott *et al.* [21], who reported V_30Gy_ in only five patients and used 3DCRT for XRT planning. Despite these differences, our findings generally align with previous reports, showing similar PT advantages for key shoulder muscles: results for the supraspinatus, latissimus dorsi, and trapezius are consistent with Burlile *et al.*, and dose differences across all seven muscles are comparable to those reported by Prescott *et al.*

Two key considerations apply to the latissimus dorsi and trapezius. For the latissimus dorsi, caution is needed due to reliance on automatic segmentation—40% of ten randomly selected cases required manual correction. For the trapezius, our V_15Gy_ [%] results differ from Burlile *et al.* [20], who reported about 30% for XRT and 10% for PT, while we found 5% for XRT and 0% for PT. This likely reflects differences in planning techniques. Overall, the trapezius shows a low dosimetric advantage of PT over Hybrid VMAT–3DCRT.

In this study, we selected dosimetric parameters with potential clinical relevance. For the five key shoulder muscles, we analysed low- and intermediate-dose metrics (V_15Gy_ and D_mean_), which have been linked to impaired arm and shoulder mobility, such as reduced abduction and lymphedema [15]. These parameters help translate dosimetric differences between XRT and PT into clinical insight: higher doses to individual muscles may impair functions like abduction, adduction, and rotation, affecting daily activities. Notably, only one clinical study on shoulder muscle dose and clinical outcomes was published in 2014, but the patients were treated more than two decades ago, highlighting the need for further investigation.

As the first study comparing XRT and PT for the ALTJ region, we found only limited overall dosimetric advantages, restricted to D_min_, even though recent publications have reported associations of D_min_, D_max_, D_mean_, and V_35Gy_ with lymphedema [13], [16], [17], [18]. Subgroup analyses showed a moderate PT advantage for D_mean_ in patients without axillary radiotherapy and for D_min_ in those treated to levels III–IV, while other parameters showed minimal or no differences. These findings suggest that ALTJ dose differences are generally small and underscore the need for larger subgroup analyses to confirm clinical relevance.

The clinical relevance of these dosimetric predictors is heightened by recent shifts in axillary management. As axillary surgery is de-escalated, indications for axillary radiotherapy are expanding, making it increasingly important to limit radiation to nearby healthy tissue, especially shoulder muscles [7], [32], [33]. In this context, our findings emphasize the value of precise axillary targeting and the potential role of PT in reducing the risk of AS.

Our study has several strengths. First, we validated shoulder muscle dose differences between XRT and PT in a relatively large cohort, including exploratory subgroup analyses for axillary radiotherapy for the first time. Second, we are the first to directly compare XRT and PT for the ALTJ region, providing novel dosimetric insights. Third, we used clinical treatment plans with muscles delineated via a standardized atlas and a hybrid segmentation strategy, which reduced contouring time, improved reproducibility, minimized observer bias, and enabled consistent cross-institutional comparisons.

Several limitations should be noted. Separate CTs for XRT and PT may have introduced variation, though mitigated by standardized setup and paired design. Dose values were not converted to equivalent dose in 2 Gy fractions, which may affect higher-dose metrics like V_35Gy_ but not V_15Gy_.

Several aspects should be addressed in future work. First, the impact of arm position on dose to individual shoulder muscles and the ALTJ region remains unclear and warrants investigation. Second, formal validation of the atlas-based delineation is ongoing and will be reported in a subsequent study. Third, it is crucial to determine whether the observed dosimetric advantages translate into clinical outcomes. Finally, randomized trials comparing proton and photon therapy, such as PARABLE [34] and RADCOMP [35], may clarify the clinical relevance of shoulder dose differences. Removed lymph nodes and BMI may influence AS and could be considered in future studies.

## Conclusion

Dose reductions were observed across all seven evaluated shoulder muscles, with the greatest benefit in the five muscles closest to the CTV. PT provided limited dosimetric advantage for the ALTJ region in the overall cohort analysis.

## Funding statement

The first author conducted this work during her PhD. She was funded by the Horizon Europe project PRE-ACT (Prediction of Radiotherapy side effects using explainable AI for patient communication and treatment modification). Ιt was supported by the European Commission through the Horizon Europe Program (Grant Agreement number 101057746), by the Swiss State Secretariat for Education, Research and Innovation (SERI) under contract number 22 00058, and by the UK government (Innovate UK application number 10061955).

## Declaration of competing interest

The authors declare that they have no known competing financial interests or personal relationships that could have appeared to influence the work reported in this paper.

## Appendix A Supplementary material

Supplementary Figure S1 Example of shoulder region delineation on two transaxial CT slices at different thoracic levels. Labeled shoulder muscles: 1) Supraspinatus; 2) Infraspinatus; 3) Subscapularis; 4) Teres minor; 5) Teres major; 6) Latissimus dorsi; 7) Trapezius; 8) ALTJ region.

Supplementary Figure S2 Example of shoulder region delineation.
