# Supplementary figures and images for: Dosimetric comparison of the shoulder region between paired photon and proton plans in breast cancer patients
Source: Tech Innov Patient Support Radiat Oncol. 2026 Mar 22;38:100393. doi: 10.1016/j.tipsro.2026.100393 (PMC13068848; doi:10.1016/j.tipsro.2026.100393)

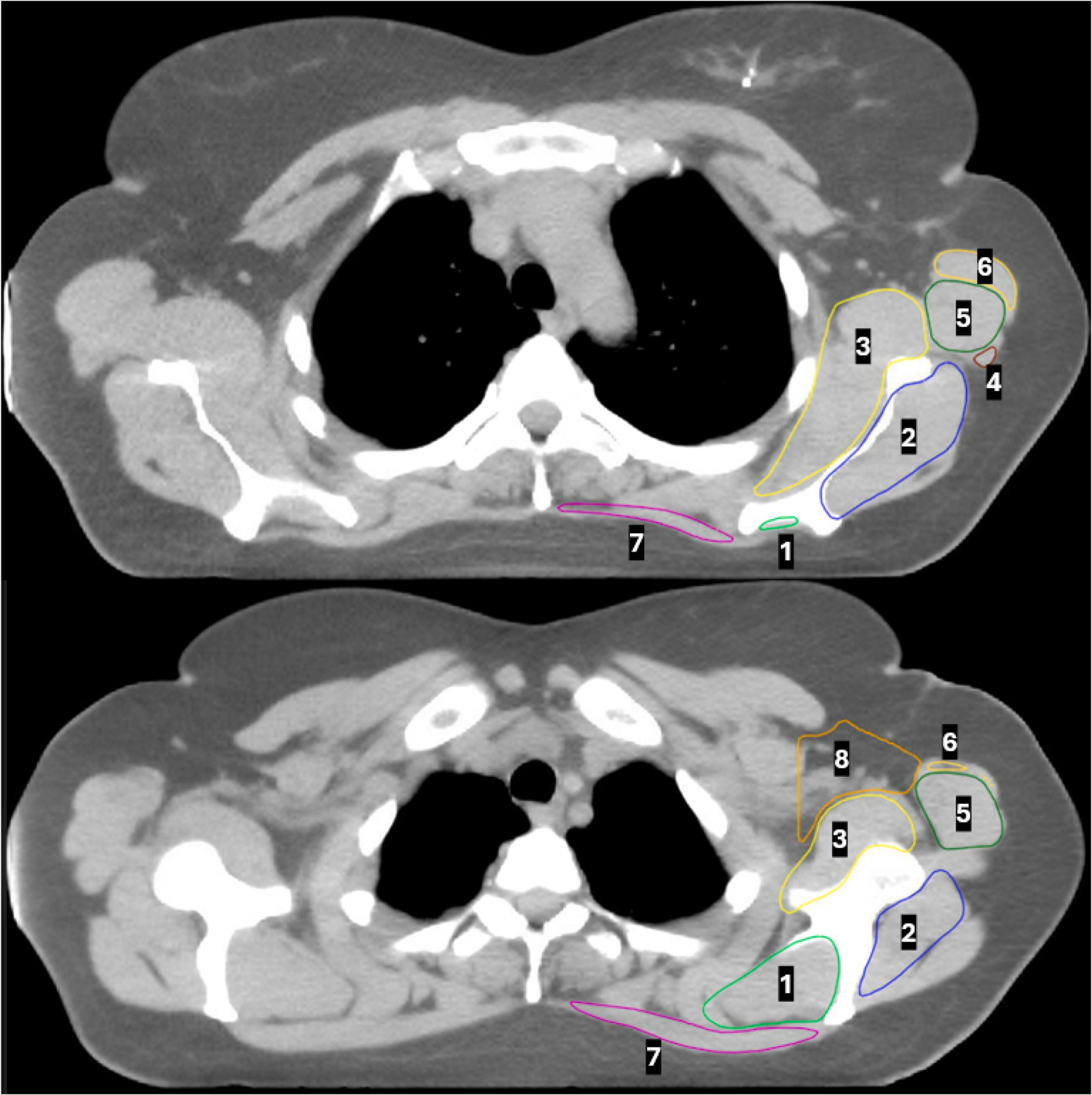

Supplement: Supplementary Figure S1 — Example of shoulder region delineation on two transaxial CT slices at different thoracic levels. Labeled shoulder muscles: 1) Supraspinatus; 2) Infraspinatus; 3) Subscapularis; 4) Teres minor; 5) Teres major; 6) Latissimus dorsi; 7) Trapezius; 8) ALTJ region. [file mmc1.jpg]

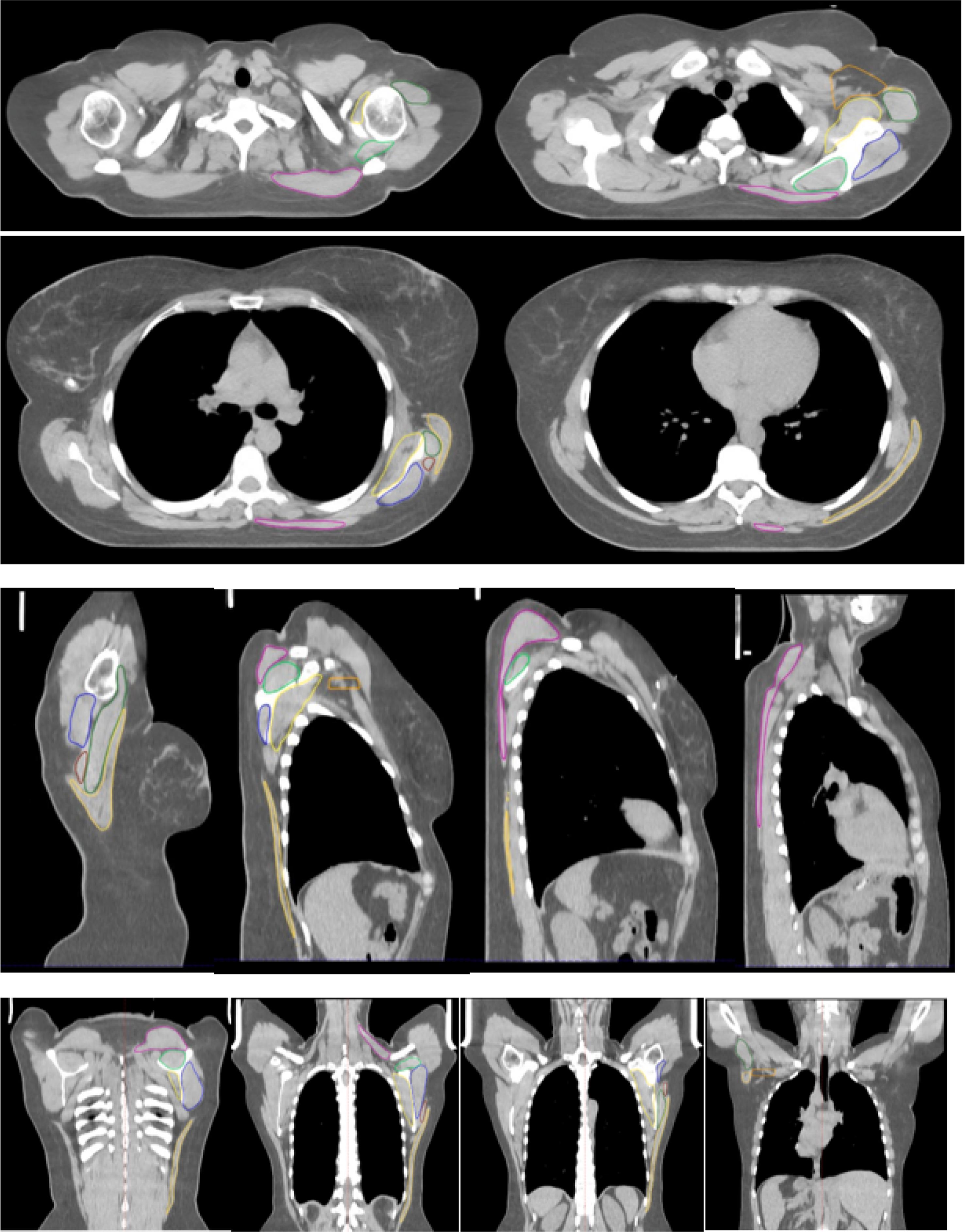

Supplement: Supplementary Figure S2 — Example of shoulder region delineation. [file mmc2.jpg]
