# Supplementary material for: Dosimetric comparison of the shoulder region between paired photon and proton plans in breast cancer patients
Source: Tech Innov Patient Support Radiat Oncol. 2026 Mar 22;38:100393. doi: 10.1016/j.tipsro.2026.100393 (PMC13068848; doi:10.1016/j.tipsro.2026.100393)
Supplement: Supplementary Data 3 [file mmc3.docx]

Supplementary Tables

| **Table A. CTV Boost Overview (n=55)** | | |
| --- | --- | --- |
| **Category** | **n (%)** | **Details** |
| Tumor bed | 39 (70.9%) | Tumor bed=39 |
| IMN | 18 (33.3%) | Lymph node in IMN (contralateral)=2; |
|  |  | Lymph node in IMN (ipsilateral)=17 |
| RNI | 9 (16.7%) | Lymph node in interpectoral space=3; |
|  |  | Lymph node in level I=4; |
|  |  | Lymph node in level II=6; |
|  |  | Lymph node in level III=5; |
|  |  | Lymph node in level IV=1 |
| Others | 4 (7.4%) | Chest wall=1; |
|  |  | Retrosternal=1; |
|  |  | Rib=1; |
|  |  | Sternum=2 |
| Abbreviations: **CTV**, clinical target volume; **IMN**, internal mammary nodes; **RNI**, regional nodal irradiation. | | |

| **Table B. Comparison of Clinical Target Volume Coverage Between XRT and PT** | | | | | | |
| --- | --- | --- | --- | --- | --- | --- |
| **All Patients** | | | | | | |
| **Structure** | **n** | **XRT median (IQR)** | **PT median (IQR)** | **median (Δᵢ)** | **HL shift (95% CI)** | **p (paired)** |
| **CTV_elective_ volume [cc]** | 128 | 628.1 (383.2–939.2) | 625.7 (377.3–921.6) | 8.8 | 15.90 (6.56 to 26.30) | <0.001 |
| **CTV_elective_ D_2%_ [Gy]** | 128 | 41.8 (41.5–54.2) | 42.1 (41.8–55) | -0.4 | -0.45 (-0.56 to -0.33) | <0.001 |
| **CTV_elective_ D_98%_ [Gy]** | 128 | 39.1 (38.6–42.2) | 39.9 (39.4–43.1) | -0.9 | -0.87 (-1.02 to -0.74) | <0.001 |
| **CTV_elective_ D_mean_ [Gy]** | 128 | 40.5 (40–45.7) | 41 (40.6–45.7) | -0.4 | -0.46 (-0.56 to -0.37) | <0.001 |
| **CTV_boost_ volume [cc]** | 55 | 49.5 (19.1–92.2) | 48.7 (16.8–87.1) | 0.3 | 1.35 (-0.48 to 4.65) | 0.140 |
| **CTV_boost_ D_2%_ [Gy]** | 55 | 55.5 (54.5–56.5) | 56.4 (55.9–56.9) | -0.9 | -0.98 (-1.29 to -0.70) | <0.001 |
| **CTV_boost_ D_98%_ [Gy]** | 55 | 52.5 (52.2–53) | 53.2 (53–53.6) | -0.7 | -0.71 (-0.85 to -0.52) | <0.001 |
| **CTV_boost_ D_mean_ [Gy]** | 55 | 54 (53.5–55.1) | 55 (54.6–55.2) | -0.8 | -0.81 (-1.06 to -0.56) | <0.001 |
| **IMN volume [cc]** | 84 | 15.9 (10.2–24.6) | 17.7 (12.3–24.3) | -1.0 | -1.15 (-2.15 to -0.10) | 0.033 |
| **IMN D_2%_ [Gy]** | 84 | 42.2 (41.3–48.5) | 42.8 (42.3–48.1) | -0.8 | -0.71 (-0.89 to -0.51) | <0.001 |
| **IMN D_98%_ [Gy]** | 84 | 39.9 (39.2–43.2) | 40.3 (39.8–43.9) | -0.5 | -0.56 (-0.76 to -0.37) | <0.001 |
| **IMN D_mean_ [Gy]** | 84 | 40.9 (40.3–45.8) | 41.5 (41–45.9) | -0.6 | -0.60 (-0.81 to -0.40) | <0.001 |
| **levels I–II volume [cc]** | 73 | 97.1 (71.1–133.5) | 97.3 (68.6–135.2) | 0.0 | -0.85 (-3.85 to 2.05) | 0.519 |
| **levels I–II D_2%_ [Gy]** | 73 | 41.5 (41–45.7) | 42 (41.8–45.4) | -0.6 | -0.49 (-0.64 to -0.32) | <0.001 |
| **levels I–II D_98%_ [Gy]** | 73 | 39.2 (38.9–42.5) | 40.2 (39.9–43.3) | -0.9 | -0.94 (-1.06 to -0.80) | <0.001 |
| **levels I–II D_mean_ [Gy]** | 73 | 40.3 (39.9–44) | 41.1 (40.9–44.4) | -0.8 | -0.78 (-0.90 to -0.65) | <0.001 |
| **levels III–IV volume [cc]** | 96 | 28.1 (21.2–43.1) | 30.9 (22.2–45.4) | -0.9 | -1.35 (-2.35 to -0.50) | 0.003 |
| **levels III–IV D_2%_ [Gy]** | 96 | 42.1 (41.6–45.9) | 42.3 (42–46.1) | -0.3 | -0.33 (-0.49 to -0.16) | <0.001 |
| **levels III–IV D_98%_ [Gy]** | 96 | 40 (39.4–42.9) | 40.4 (40–43.6) | -0.6 | -0.61 (-0.71 to -0.51) | <0.001 |
| **levels III–IV D_mean_ [Gy]** | 96 | 41 (40.6–44.7) | 41.3 (41–44.8) | -0.4 | -0.40 (-0.52 to -0.27) | <0.001 |
| **Breast volume [cc]** | 68 ^*^ | 798.9 (540.8–1024.5) | 772 (540.1–1046.2) | 16.0 | 18.99 (7.15 to 32.15) | 0.004 |
| **Breast D_2%_ [Gy]** | 68 ^*^ | 53.1 (41.5–54.5) | 54.2 (41.7–55.2) | -0.4 | -0.48 (-0.66 to -0.32) | <0.001 |
| **Breast D_98%_ [Gy]** | 68 ^*^ | 41.7 (38.6–42.3) | 42.6 (39.5–43.1) | -0.8 | -0.83 (-0.99 to -0.67) | <0.001 |
| **Breast D_mean_ [Gy]** | 68 ^*^ | 44.8 (40.2–46) | 45 (40.7–46.1) | -0.4 | -0.37 (-0.50 to -0.26) | <0.001 |
| **Chest wall volume [cc]** | 59 ^*^ | 304.5 (193.8–531.2) | 274.4 (190.4–463.2) | 16.1 | 23.45 (7.60 to 42.25) | 0.005 |
| **Chest wall D_2%_ [Gy]** | 59 ^*^ | 41.5 (41.2–46.3) | 42 (41.7–46.7) | -0.3 | -0.31 (-0.47 to -0.16) | <0.001 |
| **Chest wall D_98%_ [Gy]** | 59 ^*^ | 38.9 (38.3–39.4) | 39.6 (39–42.5) | -1.0 | -1.09 (-2.82 to -0.73) | <0.001 |
| **Chest wall D_mean_ [Gy]** | 59 ^*^ | 40.2 (39.8–44) | 40.9 (40.4–44.3) | -0.3 | -0.53 (-0.74 to -0.33) | <0.001 |
| **Left-Sided Radiotherapy** | | | | | | |
| **Structure** | **n** | **XRT median (IQR)** | **PT median (IQR)** | **median (Δᵢ)** | **HL shift (95% CI)** | **p (paired)** |
| **CTV_elective_ volume [cc]** | 99 | 690.8 (416.4–988.2) | 693.3 (387–950.3) | 14.9 | 17.35 (6.20 to 28.90) | 0.001 |
| **CTV_elective_ D_2%_ [Gy]** | 99 | 41.7 (41.4–54) | 42 (41.7–54.8) | -0.4 | -0.47 (-0.58 to -0.35) | <0.001 |
| **CTV_elective_ D_98%_ [Gy]** | 99 | 39.1 (38.6–42.1) | 39.7 (39.3–42.9) | -0.8 | -0.81 (-0.97 to -0.65) | <0.001 |
| **CTV_elective_ D_mean_ [Gy]** | 99 | 40.4 (40–45.3) | 40.9 (40.6–45.4) | -0.4 | -0.46 (-0.57 to -0.36) | <0.001 |
| **CTV_boost_ volume [cc]** | 38 | 72.9 (42.9–97.1) | 61.4 (31–91.5) | 0.8 | 3.05 (-0.28 to 10.20) | 0.070 |
| **CTV_boost_ D_2%_ [Gy]** | 38 | 55.3 (54.4–56) | 56.3 (55.8–56.5) | -1.0 | -1.00 (-1.40 to -0.61) | <0.001 |
| **CTV_boost_ D_98%_ [Gy]** | 38 | 52.4 (52–52.7) | 53.1 (52.9–53.4) | -0.8 | -0.76 (-0.95 to -0.56) | <0.001 |
| **CTV_boost_ D_mean_ [Gy]** | 38 | 53.8 (53.2–54.4) | 54.8 (54.4–55.1) | -0.9 | -0.86 (-1.19 to -0.53) | <0.001 |
| **IMN volume [cc]** | 59 | 15.9 (10.1–22) | 17 (11.8–22.8) | -1.3 | -1.25 (-2.40 to 0.00) | 0.054 |
| **IMN D_2%_ [Gy]** | 59 | 41.8 (41.2–45.8) | 42.6 (42.2–46.1) | -0.8 | -0.69 (-0.89 to -0.49) | <0.001 |
| **IMN D_98%_ [Gy]** | 59 | 39.5 (39.1–43.1) | 40.1 (39.7–43.1) | -0.5 | -0.50 (-0.69 to -0.32) | <0.001 |
| **IMN D_mean_ [Gy]** | 59 | 40.6 (40.2–44.4) | 41.3 (40.9–44.8) | -0.6 | -0.60 (-0.79 to -0.41) | <0.001 |
| **levels I–II volume [cc]** | 58 | 97.6 (71.7–133.3) | 96.2 (69.3–132.6) | -0.1 | -0.25 (-3.80 to 2.95) | 0.854 |
| **levels I–II D_2%_ [Gy]** | 58 | 41.4 (41–45.5) | 42 (41.7–45.3) | -0.6 | -0.51 (-0.67 to -0.32) | <0.001 |
| **levels I–II D_98%_ [Gy]** | 58 | 39.2 (38.8–42.2) | 40.1 (39.8–43.2) | -0.9 | -0.91 (-1.05 to -0.78) | <0.001 |
| **levels I–II D_mean_ [Gy]** | 58 | 40.2 (39.9–43.7) | 41 (40.8–44.3) | -0.8 | -0.77 (-0.89 to -0.63) | <0.001 |
| **levels III–IV volume [cc]** | 73 | 26.3 (20.6–37.6) | 28.5 (21.7–39.5) | -1.2 | -1.40 (-2.45 to -0.55) | 0.003 |
| **levels III–IV D_2%_ [Gy]** | 73 | 42 (41.6–45.4) | 42.2 (42–45.8) | -0.3 | -0.34 (-0.52 to -0.17) | <0.001 |
| **levels III–IV D_98%_ [Gy]** | 73 | 39.8 (39.4–42.9) | 40.3 (40–43.4) | -0.6 | -0.56 (-0.66 to -0.43) | <0.001 |
| **levels III–IV D_mean_ [Gy]** | 73 | 40.9 (40.5–44) | 41.2 (41–44.7) | -0.4 | -0.36 (-0.49 to -0.22) | <0.001 |
| **Breast volume [cc]** | 59 * | 803 (538.9–1030.9) | 802.4 (544.2–1070.2) | 19.0 | 21.60 (8.80 to 35.10) | 0.002 |
| **Breast D_2%_ [Gy]** | 59 * | 53.4 (41.5–54.5) | 54.5 (41.7–55.2) | -0.4 | -0.45 (-0.66 to -0.28) | <0.001 |
| **Breast D_98%_ [Gy]** | 59 * | 41.5 (38.6–42.4) | 42.5 (39.5–43.1) | -0.8 | -0.80 (-0.97 to -0.63) | <0.001 |
| **Breast D_mean_ [Gy]** | 59 * | 45 (40.2–46.1) | 45.1 (40.6–46.2) | -0.3 | -0.32 (-0.45 to -0.19) | <0.001 |
| **Chest wall volume [cc]** | 40 ^*^ | 301.6 (187.5–545.5) | 269.1 (183.1–455.8) | 17.1 | 20.84 (-0.85 to 50.00) | 0.065 |
| **Chest wall D_2%_ [Gy]** | 40 ^*^ | 41.4 (41.2–41.6) | 41.9 (41.6–42.1) | -0.5 | -0.48 (-0.59 to -0.34) | <0.001 |
| **Chest wall D_98%_ [Gy]** | 40 ^*^ | 38.7 (38–39.2) | 39.4 (39–39.8) | -0.8 | -1.04 (-7.53 to -0.52) | <0.001 |
| **Chest wall D_mean_ [Gy]** | 40 ^*^ | 40.1 (39.7–40.4) | 40.6 (40.4–41) | -0.4 | -0.63 (-0.95 to -0.37) | <0.001 |

Abbreviations: **CTV_elective_**, elective clinical target volume (whole breast/chest wall and regional nodes); **CTV_boost_**, boost clinical target volume (tumor bed or specified nodal region); **IMN**, internal mammary nodes; levels I–II, inclusion of level I–II nodal regions; levels III–IV, inclusion of level III–IV nodal regions (apical/supraclavicular); **D_2%_ [Gy]**, dose to the hottest 2% of the volume (maximum-dose proxy); **D_98%_ [Gy]**, dose to 98% of the volume (minimum-dose proxy); **D_mean_ [Gy]**, mean dose; **XRT median [IQR],** median and interquartile range (Q1–Q3) for photon therapy; **PT median [IQR],** median and interquartile range (Q1–Q3) for proton therapy; **HL shift (95% CI),** Hodges–Lehmann estimate of the paired difference (XRT − PT) with 95% confidence interval; **median (Δᵢ)**, median of within-patient paired differences for each parameter, where Δᵢ = XRTᵢ − PTᵢ for subject i; **p (paired),** Wilcoxon signed-rank test. ^*^ **Note:** One patient presented with bilateral breast cancer and consequently underwent radiotherapy to the left chest wall and the right breast. Two patients received internal mammary node–only irradiation, without irradiation of the breast chest wall, or other regional lymph node stations.

| **Table C. Subgroup analysis of dosimetric parameters for individual shoulder regions comparing photon (XRT) and proton (PT) plans.** | | | | | | | | |
| --- | --- | --- | --- | --- | --- | --- | --- | --- |
| **Subgroup** | **Dosimetric parameters** | **n** | **XRT median (IQR)** | **PT median (IQR)** | **median (Δᵢ)** | **HL shift (95% CI)** | **p (paired)** | **p (BH)** |
| All levels I–IV | Teres major D_mean_ [Gy] | 65 | 25.2 (23–27.6) | 14.4 (11.4–17.2) | 10.5 | 10.89 (9.66 to 12.18) | <0.001 | <0.001 |
| All levels I–IV | Teres major V_15Gy_ [%] | 65 | 72.4 (67.6–75.5) | 40.1 (26–51.6) | 32.6 | 33.20 (27.67 to 38.66) | <0.001 | <0.001 |
| All levels I–IV | Teres minor D_mean_ [Gy] | 65 | 26.3 (18.8–33.3) | 1.8 (0.7–2.7) | 23.8 | 23.39 (20.73 to 25.79) | <0.001 | <0.001 |
| All levels I–IV | Teres minor V_15Gy_ [%] | 65 | 83.8 (49.6–94.4) | 0 (0–0) | 73.6 | 72.75 (64.91 to 79.83) | <0.001 | <0.001 |
| All levels I–IV | Supraspinatus D_mean_ [Gy] | 65 | 18.4 (16.3–20.7) | 0.1 (0–0.3) | 17.8 | 18.00 (17.29 to 18.68) | <0.001 | <0.001 |
| All levels I–IV | Supraspinatus V_15Gy_ [%] | 65 | 68.9 (60.4–79.1) | 0 (0–0) | 67.8 | 68.34 (64.72 to 71.83) | <0.001 | <0.001 |
| All levels I–IV | Infraspinatus D_mean_ [Gy] | 65 | 14.8 (12.7–18) | 0.5 (0.2–0.9) | 14.3 | 14.55 (13.52 to 15.77) | <0.001 | <0.001 |
| All levels I–IV | Infraspinatus V_15Gy_ [%] | 65 | 46.4 (36.8–55.6) | 0 (0–0) | 46.4 | 46.76 (42.88 to 50.76) | <0.001 | <0.001 |
| All levels I–IV | Subscapularis D_mean_ [Gy] | 65 | 22 (19.3–25) | 11.7 (8.8–13.6) | 10.6 | 10.65 (9.51 to 11.88) | <0.001 | <0.001 |
| All levels I–IV | Subscapularis V_15Gy_ [%] | 65 | 63.6 (53.7–71.2) | 31.4 (24–37.6) | 30.3 | 31.54 (27.72 to 35.69) | <0.001 | <0.001 |
| All levels I–IV | Latissimus dorsi D_mean_ [Gy] | 65 | 13.3 (11.4–15.4) | 8.7 (7.1–11.4) | 4.2 | 4.28 (3.41 to 5.20) | <0.001 | <0.001 |
| All levels I–IV | Latissimus dorsi V_15Gy_ [%] | 65 | 32.9 (28–41.3) | 0 (0–0) | 31.6 | 31.74 (28.54 to 35.04) | <0.001 | <0.001 |
| All levels I–IV | Trapezius D_mean_ [Gy] | 65 | 6.2 (5.3–7.9) | 0.1 (0–0.3) | 6.0 | 6.24 (5.78 to 6.79) | <0.001 | <0.001 |
| All levels I–IV | Trapezius V_15Gy_ [%] | 65 | 11.2 (4.3–18.1) | 0 (0–0) | 10.6 | 11.67 (9.24 to 14.02) | <0.001 | <0.001 |
| All levels I–IV | ALTJ D_max_ [Gy] | 65 | 42.4 (41.8–46.3) | 42.6 (42.1–46.5) | -0.3 | -0.35 (-0.62 to -0.09) | 0.011 | 0.013 |
| All levels I–IV | ALTJ D_mean_ [Gy] | 65 | 40.2 (39.6–43.5) | 40.7 (39.7–43.5) | -0.3 | -0.21 (-0.54 to 0.17) | 0.222 | 0.235 |
| All levels I–IV | ALTJ D_min_ [Gy] | 65 | 34.3 (24.6–38.2) | 29.6 (25.1–35.8) | 1.0 | 1.61 (-0.19 to 4.05) | 0.085 | 0.096 |
| All levels I–IV | ALTJ V_35Gy_ [%] | 65 | 100 (96.8–100) | 98.8 (94.8–100) | 0.0 | 0.73 (-0.87 to 2.60) | 0.303 | 0.303 |
| Levels III–IV only | Teres major D_mean_ [Gy] | 31 | 21 (18.5–23.9) | 4.2 (1.6–6.5) | 17.3 | 17.10 (14.83 to 19.06) | <0.001 | <0.001 |
| Levels III–IV only | Teres major V_15Gy_ [%] | 31 | 64.7 (58.3–75.3) | 3.1 (0–13.2) | 57.9 | 57.74 (51.45 to 62.69) | <0.001 | <0.001 |
| Levels III–IV only | Teres minor D_mean_ [Gy] | 31 | 21.9 (15–29.7) | 0.5 (0.2–1) | 20.8 | 21.62 (17.73 to 25.89) | <0.001 | <0.001 |
| Levels III–IV only | Teres minor V_15Gy_ [%] | 31 | 69 (45.6–98.3) | 0 (0–0) | 69.0 | 71.51 (55.75 to 81.96) | <0.001 | <0.001 |
| Levels III–IV only | Supraspinatus D_mean_ [Gy] | 31 | 17.3 (14.5–20.2) | 0.1 (0–0.3) | 17.2 | 17.10 (15.62 to 18.40) | <0.001 | <0.001 |
| Levels III–IV only | Supraspinatus V_15Gy_ [%] | 31 | 66.1 (50.3–80.5) | 0 (0–0) | 66.1 | 65.06 (56.20 to 72.76) | <0.001 | <0.001 |
| Levels III–IV only | Infraspinatus D_mean_ [Gy] | 31 | 13.1 (9.9–16.4) | 0.2 (0.1–0.2) | 12.9 | 13.10 (11.26 to 15.55) | <0.001 | <0.001 |
| Levels III–IV only | Infraspinatus V_15Gy_ [%] | 31 | 40.2 (27.8–54.9) | 0 (0–0) | 40.2 | 41.62 (34.40 to 50.09) | <0.001 | <0.001 |
| Levels III–IV only | Subscapularis D_mean_ [Gy] | 31 | 19.7 (16.5–23.3) | 5.7 (3.5–8.8) | 13.8 | 14.06 (11.78 to 16.36) | <0.001 | <0.001 |
| Levels III–IV only | Subscapularis V_15Gy_ [%] | 31 | 65.5 (52.3–73.1) | 13.6 (7–23.6) | 47.7 | 47.42 (39.45 to 54.89) | <0.001 | <0.001 |
| Levels III–IV only | Latissimus dorsi D_mean_ [Gy] | 31 | 16 (12.2–19.6) | 5.8 (3.1–9.2) | 9.1 | 8.87 (6.29 to 12.06) | <0.001 | <0.001 |
| Levels III–IV only | Latissimus dorsi V_15Gy_ [%] | 31 | 40.7 (32.9–53.1) | 0 (0–0) | 40.1 | 39.63 (34.27 to 45.28) | <0.001 | <0.001 |
| Levels III–IV only | Trapezius D_mean_ [Gy] | 31 | 7.2 (5.7–8.1) | 0.1 (0–0.4) | 6.8 | 6.55 (5.79 to 7.14) | <0.001 | <0.001 |
| Levels III–IV only | Trapezius V_15Gy_ [%] | 31 | 10 (2.2–17.6) | 0 (0–0) | 10.0 | 10.44 (7.65 to 14.78) | <0.001 | <0.001 |
| Levels III–IV only | ALTJ D_max_ [Gy] | 31 | 42.2 (41.6–46.2) | 42.8 (41.8–46) | -0.0 | -0.09 (-0.58 to 0.47) | 0.766 | 0.766 |
| Levels III–IV only | ALTJ D_mean_ [Gy] | 31 | 37.5 (32–39.5) | 34.2 (27.8–39.4) | 0.9 | 1.64 (0.04 to 4.12) | 0.035 | 0.040 |
| Levels III–IV only | ALTJ D_min_ [Gy] | 31 | 19.7 (14.1–25) | 9 (4.3–17.5) | 9.4 | 9.08 (6.09 to 10.99) | <0.001 | <0.001 |
| Levels III–IV only | ALTJ V_35Gy_ [%] | 31 | 69.6 (34.5–88.8) | 61.3 (31.7–85.3) | 3.5 | 3.56 (-1.57 to 9.22) | 0.139 | 0.147 |
| Levels I–II only | Teres major D_mean_ [Gy] | 8 | 25.2 (24.5–27.1) | 14.4 (13–16.8) | 12.2 | 11.42 (9.42 to 12.86) | 0.014 | 0.024 |
| Levels I–II only | Teres major V_15Gy_ [%] | 8 | 70.6 (67.5–73.3) | 42.3 (34.7–50.6) | 28.8 | 27.46 (18.92 to 52.01) | 0.014 | 0.024 |
| Levels I–II only | Teres minor D_mean_ [Gy] | 8 | 22.5 (16.4–32.8) | 2.6 (1.4–4) | 19.0 | 19.95 (10.15 to 30.72) | 0.014 | 0.024 |
| Levels I–II only | Teres minor V_15Gy_ [%] | 8 | 71 (38.9–100) | 0 (0–0) | 71.0 | 60.97 (34.42 to 100.00) | 0.014 | 0.024 |
| Levels I–II only | Supraspinatus D_mean_ [Gy] | 8 | 3.7 (2.2–3.8) | 0 (0–0.1) | 3.5 | 3.26 (2.12 to 4.67) | 0.014 | 0.024 |
| Levels I–II only | Supraspinatus V_15Gy_ [%] | 8 | 0 (0–0) | 0 (0–0) | 0.0 | 0.50 (0.01 to 1.25) | 0.181 | 0.240 |
| Levels I–II only | Infraspinatus D_mean_ [Gy] | 8 | 10.5 (9.2–12.8) | 0.9 (0.5–1.2) | 9.2 | 9.75 (6.74 to 12.93) | 0.014 | 0.024 |
| Levels I–II only | Infraspinatus V_15Gy_ [%] | 8 | 23.3 (18.5–30.2) | 0 (0–0) | 23.2 | 23.82 (14.32 to 38.04) | 0.014 | 0.024 |
| Levels I–II only | Subscapularis D_mean_ [Gy] | 8 | 19.8 (15.6–20) | 13.1 (11.2–13.9) | 5.6 | 5.01 (0.23 to 9.29) | 0.042 | 0.065 |
| Levels I–II only | Subscapularis V_15Gy_ [%] | 8 | 48.3 (37–51.3) | 36.3 (30.7–40.6) | 8.7 | 8.59 (-7.09 to 23.50) | 0.234 | 0.284 |
| Levels I–II only | Latissimus dorsi D_mean_ [Gy] | 8 | 12.3 (11–14.3) | 8.3 (6.6–9.6) | 3.9 | 3.70 (1.36 to 7.78) | 0.014 | 0.024 |
| Levels I–II only | Latissimus dorsi V_15Gy_ [%] | 8 | 30.8 (26.6–37.1) | 0 (0–5.7) | 26.3 | 24.43 (12.16 to 35.05) | 0.014 | 0.024 |
| Levels I–II only | Trapezius D_mean_ [Gy] | 8 | 1.1 (0.7–1.2) | 0 (0–0) | 1.1 | 0.97 (0.60 to 1.30) | 0.014 | 0.024 |
| Levels I–II only | Trapezius V_15Gy_ [%] | 8 | 0 (0–0) | 0 (0–0) | 0.0 | * | * | * |
| Levels I–II only | ALTJ D_max_ [Gy] | 8 | 41.1 (40.9–41.6) | 42.4 (42.2–42.6) | -1.4 | -1.30 (-1.75 to 1.64) | 0.183 | 0.240 |
| Levels I–II only | ALTJ D_mean_ [Gy] | 8 | 39.6 (36.9–39.9) | 37.3 (35.7–40.3) | -0.5 | -0.48 (-4.23 to 4.82) | 0.944 | 0.944 |
| Levels I–II only | ALTJ D_min_ [Gy] | 8 | 35.6 (26.9–37.3) | 22.4 (17–31.6) | 2.5 | 5.38 (-8.68 to 19.14) | 0.441 | 0.500 |
| Levels I–II only | ALTJ V_35Gy_ [%] | 8 | 100 (87.2–100) | 74.9 (63.1–99.3) | 0.5 | 6.31 (-29.14 to 41.96) | 0.554 | 0.589 |
| No levels I–IV | Teres major D_mean_ [Gy] | 24 | 15.8 (8.3–21) | 1.3 (0.5–2.7) | 13.9 | 12.45 (8.92 to 15.98) | <0.001 | <0.001 |
| No levels I–IV | Teres major V_15Gy_ [%] | 24 | 40.1 (20.7–59.9) | 0 (0–0) | 38.1 | 44.91 (34.60 to 54.35) | <0.001 | <0.001 |
| No levels I–IV | Teres minor D_mean_ [Gy] | 24 | 7 (2.6–16.3) | 0.3 (0.1–0.5) | 6.9 | 9.71 (5.16 to 14.23) | <0.001 | <0.001 |
| No levels I–IV | Teres minor V_15Gy_ [%] | 24 | 10.1 (0.1–44.3) | 0 (0–0) | 10.1 | 36.12 (18.31 to 54.95) | <0.001 | <0.001 |
| No levels I–IV | Supraspinatus D_mean_ [Gy] | 24 | 0.7 (0.4–1.5) | 0 (0–0) | 0.7 | 0.86 (0.57 to 2.08) | <0.001 | <0.001 |
| No levels I–IV | Supraspinatus V_15Gy_ [%] | 24 | 0 (0–0) | 0 (0–0) | 0.0 | 2.45 (2.44 to 2.44) | 0.371 | 0.393 |
| No levels I–IV | Infraspinatus D_mean_ [Gy] | 24 | 2.4 (1.3–5.8) | 0 (0–0.1) | 2.4 | 3.25 (1.87 to 5.92) | <0.001 | <0.001 |
| No levels I–IV | Infraspinatus V_15Gy_ [%] | 24 | 0.1 (0–11.8) | 0 (0–0) | 0.1 | 11.55 (2.64 to 27.40) | 0.002 | 0.002 |
| No levels I–IV | Subscapularis D_mean_ [Gy] | 24 | 4.8 (2.6–9.5) | 0.3 (0.1–0.5) | 3.5 | 4.83 (2.87 to 8.13) | <0.001 | <0.001 |
| No levels I–IV | Subscapularis V_15Gy_ [%] | 24 | 6.6 (0.3–24.7) | 0 (0–0) | 5.0 | 14.91 (4.97 to 28.24) | <0.001 | <0.001 |
| No levels I–IV | Latissimus dorsi D_mean_ [Gy] | 24 | 9.1 (6.3–11) | 3.3 (1.8–5.7) | 4.9 | 4.45 (2.06 to 6.53) | <0.001 | 0.001 |
| No levels I–IV | Latissimus dorsi V_15Gy_ [%] | 24 | 22.8 (14.4–28) | 0 (0–1.4) | 19.6 | 20.25 (13.49 to 26.05) | <0.001 | <0.001 |
| No levels I–IV | Trapezius D_mean_ [Gy] | 24 | 0.3 (0.3–0.9) | 0 (0–0) | 0.3 | 0.53 (0.31 to 0.90) | <0.001 | <0.001 |
| No levels I–IV | Trapezius V_15Gy_ [%] | 24 | 0 (0–0) | 0 (0–0) | 0.0 | * | 1.000 | 1.000 |
| No levels I–IV | ALTJ D_max_ [Gy] | 24 | 34.9 (21–40.1) | 25.6 (13.2–37.4) | 3.1 | 4.41 (1.29 to 7.78) | 0.011 | 0.013 |
| No levels I–IV | ALTJ D_mean_ [Gy] | 24 | 19.7 (6.7–27.8) | 6.7 (2.4–11.4) | 8.0 | 9.61 (5.74 to 13.23) | <0.001 | <0.001 |
| No levels I–IV | ALTJ D_min_ [Gy] | 24 | 2.9 (1.2–10.4) | 0 (0–0.2) | 2.8 | 4.96 (1.93 to 8.22) | <0.001 | <0.001 |
| No levels I–IV | ALTJ V_35Gy_ [%] | 24 | 0 (0–5.1) | 0 (0–0.6) | 0.0 | 9.16 (0.47 to 25.87) | 0.025 | 0.029 |

**Abbreviations:** **levels I–II only**, inclusion of level I–II nodal regions; **levels III–IV only**, inclusion of level III–IV nodal regions; **all levels I–IV**, inclusion of level I–IV nodal regions; **no levels I–IV**, exclusion of all levels I–IV nodal regions; **No levels I–IV**, no regional nodal irradiation (levels I–IV); **ALTJ**, axillary–lateral thoracic vessel juncture; **XRT**, photon therapy; **PT**, proton therapy; **D_mean_ [Gy]**, mean dose; **D_min_ [Gy]**, minimum dose; **D_max_ [Gy]**, maximum dose; **V_15Gy_ [%]**, percentage of the structure volume receiving ≥15 Gy; **V_35Gy_ [%]**, percentage of the structure volume receiving ≥35 Gy; **XRT median [IQR],** median and interquartile range (Q1–Q3) for photon therapy; **PT median [IQR],** median and interquartile range (Q1–Q3) for proton therapy; **HL shift (95% CI),** Hodges–Lehmann estimate of the paired difference (XRT − PT) with 95% confidence interval; **median (Δᵢ)**, median of within-patient paired differences for each parameter, where Δᵢ = XRTᵢ − PTᵢ for subject i; **p (paired),** Wilcoxon signed-rank test; **p (BH),** Benjamini–Hochberg adjusted p value; When within-patient paired differences show no or insufficient variability, the Wilcoxon signed-rank test and/or the Hodges–Lehmann shift estimate (95% CI) are not defined; in such cases, corresponding p values or HL estimates are not reported. P values equal to 1.000 indicate minimal but non-zero variability in paired differences. Colour coding: green indicates high advantage for shoulder muscles (both D_mean_ and V_15_ ≥ 10). If only one parameter exceeds the threshold (either D_mean_ or V_15_ ≥ 10), the result is considered a single-parameter advantage and is not coloured. If neither exceeds the threshold, no advantage is present. Uncoloured rows may still have significant p values, indicating differences that do not fulfil the combined threshold definition. All ALTJ results are shown without colour, as none met the predefined criteria for high advantage (*i.e.*, any of D_max_, D_mean_, D_min_, or V_35_ ≥ 10).
